# P3b Does Not Reflect Perceived Contrasts

**DOI:** 10.1523/ENEURO.0387-21.2022

**Published:** 2022-04-05

**Authors:** Yen-Kuang Chen, Tony Cheng, Po-Jang Hsieh

**Affiliations:** 1 Department of Psychology, National Taiwan University, Taipei City 10617, Taiwan; 2 Department of Philosophy, National Chengchi University, Taipei City 116302, Taiwan

**Keywords:** attention, consciousness, ERP, neural correlate of consciousness, no-report paradigm, P3b

## Abstract

It has been shown that P3b is not a signature of perceptual awareness per se but is instead more closely associated with postperceptual processing (Cohen et al., 2020). Here, we seek to investigate whether human participants’ attentional states are different in the report and the no-report conditions. This difference in attentional states, if exists, may lead to degraded consciousness of the stimuli in the no-report condition, and it therefore remains unknown whether the disappearance of P3b is because of a lack of reportability or degraded consciousness. Results of our experiment 1 showed that participants did experience degraded contents of consciousness in the no-report condition. However, results of experiment 2 showed that the degraded contents of consciousness did *not* influence the amplitude of P3b. These findings strengthen the claim that P3b is *not* a signature of perceptual awareness but is associated with postperceptual processing.

## Significance Statement

P3b, one of the most studied event-related potentials, has been claimed to reflect several cognitive activities, including consciousness. Although recent experiments with the new no-report paradigms suggest otherwise that P3b may not reflect human conscious activities, these results are not yet conclusive. In this study, we conducted a series of modified no-report paradigm experiments, and found that P3b was not modulated by the degrees of conscious perception of stimuli. These results support the claims that P3b may not be a neural marker of consciousness.

## Introduction

The debate about the exact location of the neural correlates of consciousness (NCCs) in our brain has lasted for decades. Some studies reported sensory NCCs in the anterior brain regions (Lumer and Rees, 1999; Dehaene and Naccache, 2001; Lau and Rosenthal, 2011; Cohen et al., 2012; Panagiotaropoulos et al., 2012; Noy et al., 2015; Huth et al., 2016; Dehaene et al., 2017; Siclari et al., 2017; Brown et al., 2019), while others hold that NCCs in the posterior brain regions only (Penfield and Jasper, 1954; Damasio and Van Hoesen, 1983; George et al., 1996; Markowitsch and Kessler, 2000; Mataró et al., 2001; Farah, 2004; Brown et al., 2010; Craig, 2010; von Arx et al., 2010; Barton, 2011; Quiroga, 2012; Seth et al., 2012; Kozuch, 2014; Koch et al., 2016). Many NCCs were found by using contrastive analysis (Baars, 1994) that suffered from a thorny methodological problem: most paradigms of NCCs require participants to report whether they consciously perceive the stimuli. Thus, it is difficult to tease apart the NCC and the neural correlates of reports, and the so-called “NCCs” might reflect other cognitive processes than consciousness per se (Overgaard, 2004; Block, 2007; de Graaf et al., 2012; Neisser, 2012).

To address this issue, a variety of no-report paradigms have been developed in recent studies (Frässle et al., 2014; Shafto and Pitts, 2015; Koch et al., 2016; Pitts et al., 2018; Hesse and Tsao, 2020). Participants in no-report tasks are typically not asked to report during the stimuli-presentation phase and hence do not need to make any judgment about their conscious perceptual experiences. With these paradigms, neural activities related to postperceptual judgments are arguably minimized when comparing the conscious and unconscious conditions.

Using such a no-report paradigm, a recent study showed that P3b disappeared in the no-report condition but can still be observed in the report condition (Cohen et al., 2020). Based on this result, Cohen et al. (2020) claimed that “the P3b is not a signature of perceptual awareness per se and is instead more closely associated with postperceptual processing.” However, there is strong evidence that the shifting of spatial attention can alter observers’ perceived visual appearances, such as contrast, spatial, color, and temporal dimension, etc. (Carrasco and Barbot, 2019). We therefore suspect that participants’ attentional states may not be equal in the report and the no-report conditions, which may lead to more degraded consciousness of the stimulus in the no-report condition than in the report condition. Hence, it is unclear whether the disappearance of P3b was correlated with the reportability or the degraded consciousness.

We hypothesized in experiment 1a that, compared with the report condition, participants would experience degraded consciousness of the stimuli in the no-report condition because of a lack of attention. This hypothesis is supported by the results of experiment 1a: participants reported that they perceived lower-contrast stimuli in the no-report condition. Next, experiment 1b was conducted to control for the order/learning effect, and the results successfully ruled out this potential confounding factor.

To directly examine whether degraded consciousness of the stimuli modulates P3b, we manipulated the perceived contrasts of the experimental stimuli (i.e., conscious contents) and measured P3b in experiment 2. We reasoned that if P3b really reflects perceived stimulus contrast, differences in P3b should be observable while participants perceive higher-contrast stimuli versus lower-contrast stimuli. Otherwise, we should observe a similar P3b pattern while participants perceive different contrast levels of the stimuli. Our results showed a similar P3b pattern across conditions of different perceived contrasts, and therefore are in line with Cohen et al. (2020) supporting the claim that P3b may be more closely associated with postperceptual processing.

## Materials and Methods

### Experiment 1a, behavioral experiment

The experimental design was closely modeled on the no-report paradigm of Cohen et al. (2020) to investigate whether the perceived contrast was lower in the no-report condition.

#### Participants

Forty-eight young adults took part in this experiment (12 males). All of them were recruited within the National Taiwan University community with ages from 18 to 30 years (mean age = 21.3 years), had normal or corrected to normal vision, reported no neurologic and psychiatric disorders and received pecuniary remuneration after finishing the tasks. Three participants were excluded from the analyses because of outlier performances (out of 2 SEs from the average performances). This work was approved by the ethics committee of behavioral and social science at National Taiwan University and was conducted according to its guidelines. Before the experiment, the written informed consent was given by all participants.

#### Experimental design

A mixed model design for three-factors (between-between-within design) was adopted. The first factor (between) was the pop-out times of green ring (Times): 4, 6, and 8. The second factor (between) was the contrast levels of the Gabor gratings at the beginning in the incidental memory task (Levels): 0.3 and 0.9. The contrast level was defined by the visibility of the stimulus relative to the white background, ranging from 0.0 (transparent) to 1.0 (operant). Table 1 shows the corresponding relation between the Weber Contrast and the contrast defined in this study. The third factor (within) was the report/no-report condition (R/NR). A total of 48 participants were randomly assigned to one of the six groups (Times × Level = 6; Table 2).

**Table 1:** Corresponding relation between Weber contrast and contrast in this study

Contrast level	Weber contrast
0	0
0.1	0.09
0.2	0.23
0.3	0.41
0.4	0.57
0.5	0.71
0.6	0.95
0.7	1.15
0.8	1.35
0.9	1.52
1	1.87

**Table 2:** Table of the experimental design

Conditions			
Times	Levels	No-report	Report
4	0.3	*N* = 8	
	0.9	*N* = 8	
6	0.3	*N* = 8	
	0.9	*N* = 8	
8	0.3	*N* = 8	
	0.9	*N* = 8	

A mixed model design for three-factors. Times: pop-out times of green ring; Levels: the contrast levels of the Gabor gratings at the beginning in the incidental memory task; *N*: numbers of the participants. A total of 48 participants were divided into six groups, which is the combination of the three levels in Times and the two levels in Levels. All participants completed both no-report condition and report condition.

**Figure 1.**
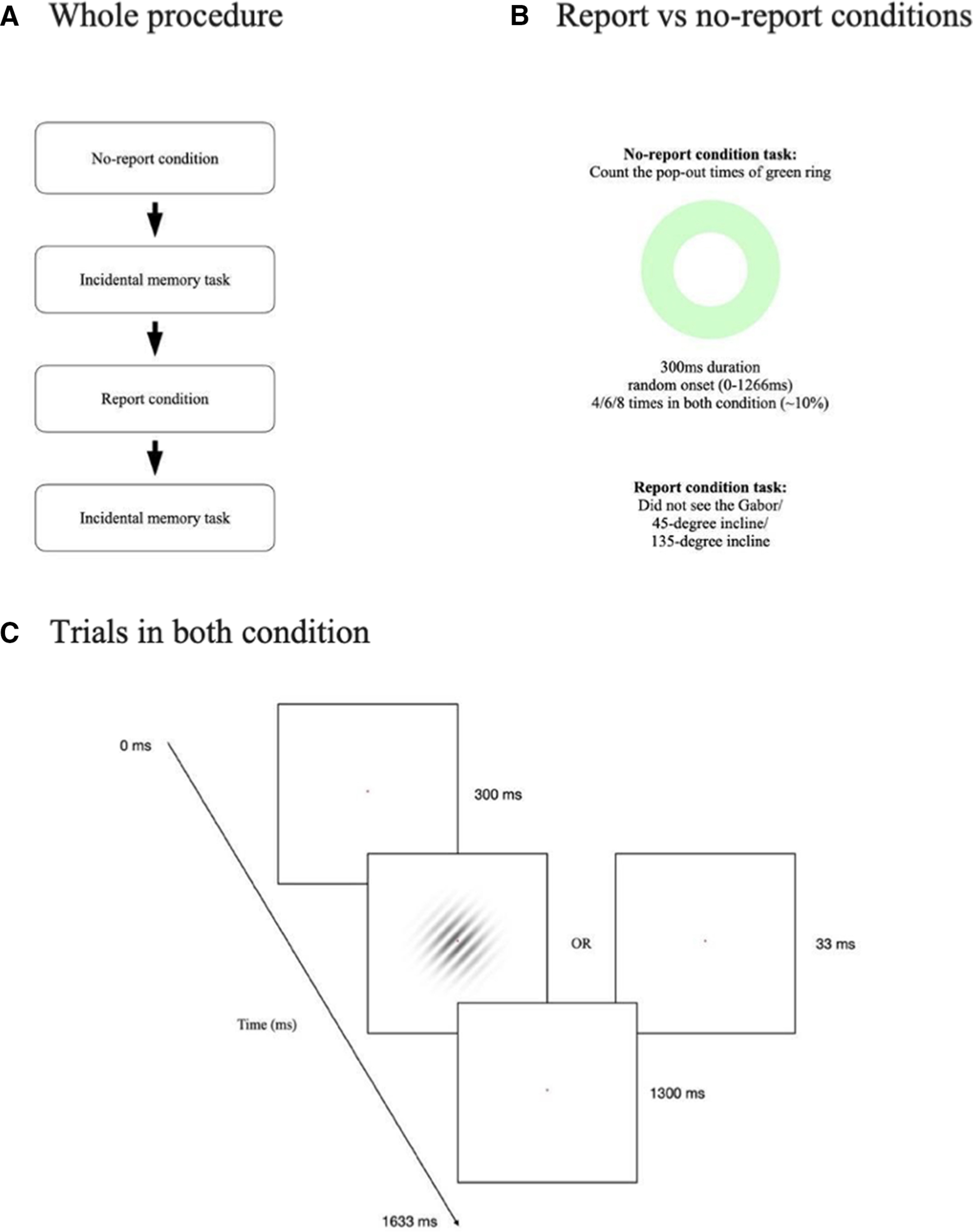
Design of the experiment. ***A***, Each participant completed this experiment in the same sequence. ***B***, In the no-report condition, the green ring was the target. Participants had to report how many times they saw the green ring. In the report condition, participants answered questions with regard to the Gabor grating they saw trial by trial. ***C***, Each trial started with 300 ms of fixation followed by 33 ms of the stimulus, and then 1300 ms of ITI. ITI, Intertrial interval.

The procedure of the experiment is shown in Figure 1. Each participant completed all four phases of the experimental procedure in a soundproof room with controlled illumination. All of them completed the experiment in a fixed order: (1) the no-report condition with 64 trials; (2) an incidental memory task on the critical stimuli; (3) the report condition with 64 trials; (4) an incidental memory task on the critical stimuli.

The no-report condition consisted of 32 critical trials and 32 blank trials with randomized sequence. Each critical trial began with a red dot at the center of the screen for 300 ms, followed by a Gabor grating with 45° incline or 135° incline for 33 ms, and then a white background for 1300 ms as the intertrial interval (ITI). The Gabor gratings were 300 × 300 pixels (px) in size with a Gaussian mask: spatial frequency = 0.035 (10.5 cycles across 300 px; opacity = 0.6; 0 as transparent and 1 as opaque). In blank trials, the 33-ms Gabor grating was replaced with a blank screen. A green ring appeared in some of the trials and served as the target in the no-report condition. The green (RGB: 196, 255, 199) ring was a circle dugout with a smaller circle from the center (longer diameter = 750 px; shorter diameter = 400 px). The onset of the green ring within a trial was selected randomly from 0 to 1266 ms, and the duration was always 300 ms. The pop-out times of green ring (the number of trials containing a green ring) depended on which group the participants were assigned (4, 6, 8 times, respectively). Participants in this condition were instructed to fixate at the red dot at the center of the screen and count how many times they see a green ring. After the participants passively viewed these 64 trials (∼106.6 s in total), they were unexpectedly asked to report the pop-out times of the green ring.

In the incidental memory task, participants was presented with the same stimuli as in the no-report condition except that the contrasts of the Gabor gratings were different. The contrast of Gabor gratings in both the report and the no-report conditions were always 0.6. However, in the incidental memory task, the Gabor gratings had an initial contrast of either 0.3 or 0.9. Participants were instructed to adjust the contrast of the Gabor gratings to match what they just saw in the previous phase (i.e., in the no-report or the report condition).

In the report condition, the stimuli and procedure were identical to those in the no-report condition (Fig. 1*C*) except the participants were instructed to respond to the Gabor gratings rather than the green rings. Participants were asked to provide a 3-AFC button-press response which indicated whether they saw a “Gabor grating inclined to 45 degrees,” a “Gabor grating inclined to 135 degrees,” or “nothing” in each trial. The green rings were still randomly presented in some trials, and the pop-out times depended on which group the participants were assigned. However, participants were not asked to report anything about the green rings.

All the experimental stimuli were controlled using Psychopy version 3.0, a psychology software in Python. All of the stimuli were displayed on a white background and located at the center of the screen. Participants perceived the stimuli with their chin resting on a chin rest to keep visual angles the same. Distance between the participant’s eyes and the monitor was 78.5 cm, making the critical stimuli 6.27°, and the green ring 15.88°. Also, participants were asked to maintain fixation on a red dot with a visual angle of 0.31° in the center of the screen throughout the whole procedure.

#### Statistical analysis

Statistical analyses were performed in R 4.0.4, using packages tidyverse, ggpubr, rstatix, and all the respective dependencies. The significance level was set at *p *=* *0.05, and generalized η^2^ was used as a measure of effect size. The null hypothesis was that there were no main effects in Times, Levels, R/NR factors.

### Experiment 1b, behavioral experiment

In this experiment, the same report condition was repeated twice to investigate whether there is a sequence effect that might influence participants’ performances in the two incidental memory tasks.

#### Participants

Forty-eight young adults took part in this experiment (12 males). They were recruited within the National Taiwan University community and voluntarily participated in this experiment with ages from 18 to 30 years (mean age = 21.4 years). Four participants were excluded from the analyses because of outlier performances (the difference of reported contrast levels between the first and the second report conditions were out of 2 SEs from the group mean).

#### Visual stimulation and experimental design

All stimuli and procedures were identical to those in experiment 1a except that there was no no-report condition in this experiment. Participants were asked to complete two phases of report condition and two phases of incidental memory task in a fixed order: (1) the first report condition with 64 trials; (2) an incidental memory task on the critical stimuli; (3) the second report condition with 64 trials; (4) an incidental memory task on the critical stimuli.

### Experiment 2

#### EEG experiment

In the behavioral experiment, the results supported our hypothesis that participants in the no-report condition perceived lower contrasts of the stimuli than in the report condition. We further conducted an EEG experiment to investigate whether the difference between these two conditions affected the amplitude of the P3b component.

#### Participants

Twenty-two young adults took part in this experiment (12 males) with ages from 20 to 33 years (mean age = 26.0 years). They were recruited within the National Taiwan University community and voluntarily participated in this experiment. All of them reported being free of neurologic and psychiatric disorders and received pecuniary remuneration after finishing the tasks. The participants were also informed to avoid caffeine-containing drinks and any kind of medicine a few days before the experiment. Two participants were excluded from the analyses (one because of the hardware-facility problem and the other one because of high artifacts).

#### Apparatus

The data were recorded with a 32 Channel EEG Quick-Cap connected to a NuAmps monopolar digital amplifier (a 40-channel-EEG-ERP-amplifier, Neuroscan) and a computer running SCAN 4.5 software. Electrode positions on the Quick-Cap follow the International 10–20 system. Electrode impedances were kept below 10 kV and bandpass filtered from 0.1 to 150 Hz. Eye movements were monitored by left and right horizontal EOG channels, and the blinks were recorded by two vertical EOG electrodes (one above and the other below the left eye).

#### Visual stimulation and experimental design

All stimuli and procedures were identical to those in experiment 1a except for the following. Each participant completed all six phases of the experimental procedure in a fixed order: (1) six no-report blocks with 64 trials in each block; (2) an incidental memory task on the critical stimuli in the previous no-report blocks; (3) six report blocks with 64 trials in each block; (4) an incidental memory task on the critical stimuli in the previous report blocks; (5) six report blocks with 64 trials in each block with reduced contrasts of critical stimuli comparing to those in the first and the third phases (reduced from 0.6 to 0.3); (6) an incidental memory task on the critical stimuli in the last six report blocks.

For the six blocks in the no-report and the report conditions, two contained four pop-out times of green ring, another two contained six times, and the other two contained eight times, making the pop-out times of green ring around ten percent of the trials on average. The sequence of the blocks was randomized. Participants with odd subject numbers were assigned to the 0.3 Levels group, and those with even subject numbers were assigned to the 0.9 Levels group. Also, the orders of critical trials and blank trials were randomized in the incidental memory tasks. Trial structures were identical to those in Experiment 1a except that each trial started with a red dot with a duration randomly jittered from 200 to 400 ms, and the ITI was lengthed to 1633 ms.

#### Statistical analyses

The EEG data were recorded at a sampling rate of 1000 Hz and processed with the Brainstorm software package (Tadel et al., 2011). Recordings were low-pass filtered at 25 Hz with a 24 dB/Oct roll-off, and referenced to the average of the left and right mastoids. The independent component analysis (ICA) was adopted for rejecting the artifacts, and the ICA components were sorted based on their correlation with the EOGs and ECG channels. ICA components with their topographies reflected artifacts such as blinks, eye movements, muscle activities, and heartbeats were removed. Artifacts not cleaned by the filter and ICA cleaning were marked as bed segments for rejecting the “Detect other artifacts” function in the Brainstorm and further removed manually. On average, 7.2% of trials were excluded because of artifacts among recruited participants, leaving on average around 178 trials for analysis per condition.

ERPs were time-locked to the onset of critical stimulus or blank in each trial. The baselines were corrected from −200 to 0 ms. Time windows and electrodes for statistical analyses were based on former studies using similar targets (Gabor gratings) or tasks. The spatial-temporal regions (ROIs) of interest for the P3b is between 300 and 400 ms (Marzecová et al., 2017; Barceló and Cooper, 2018; Brydges and Barceló, 2018). Since P3b is a component usually observed in the posterior brain area (Schröder et al., 2016; Porcaro et al., 2019), we quantified the P3b component in each condition by computing the mean voltage from 300 to 400 ms in a pool of three electrodes centered around Pz (Pz, P3, P4) for the following hypothesis-driven statistical analyses (Sardari et al.,2019).

The behavioral analyses were performed in R 4.0.4, using packages tidyverse, ggpubr, rstatix, and all the respective dependencies. The significance level was set at *p *=* *0.05, and generalized η^2^ was used as a measure of effect size. The null hypothesis was specified as that there is no difference between results of the first incidental memory task and the second incidental memory task, and no main effect in the Levels factor.

## Results

### Experiment 1a

**Figure 2.**
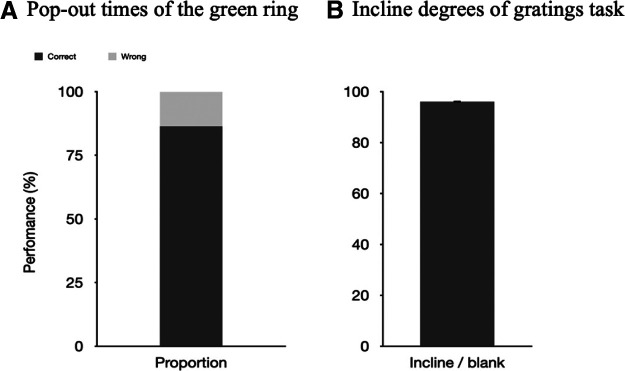
Results from both conditions in experiment 1a. The percentage correct (i.e., performance) is plotted on the *y*-axis. ***A***, The proportion that the participants correctly counted the pop-out times of the green ring. ***B***, Performance on the Gabor grating task in the report condition. The error bar indicates the SE (0.097%). SE, Standard error.

In the no-report condition, 86.36% of the participants correctly reported the pop-out times of green rings (Fig. 2*A*). The results of the incidental memory tasks showed that the mean value of perceived contrast of the Gabor gratings for the participants was 0.326. In the report condition, participants correctly reported 96.02% (SD = 0.097%) of trials (Fig. 2*B*). In the subsequent incidental memory task, the mean value of perceived contrast of the Gabor gratings was 0.566.

**Figure 3.**
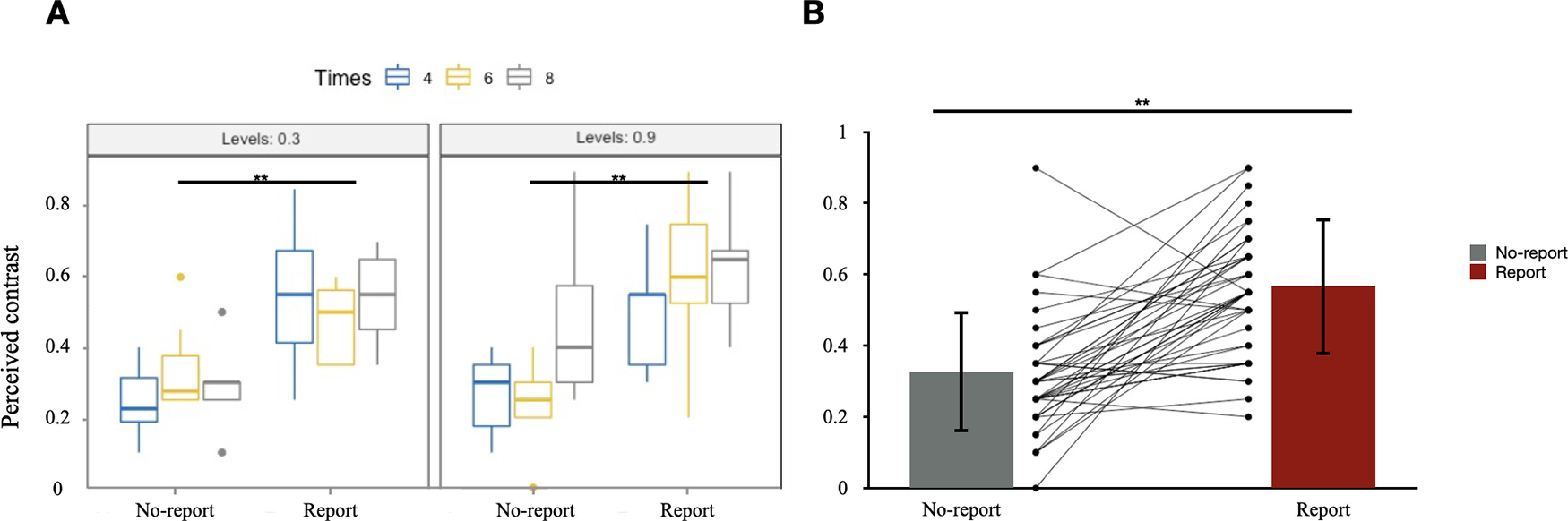
***A***, Perceived contrasts in the incidental memory tasks within all conditions. Three levels in Times: 4, 6, 8; two levels in Levels: 0.3, 0.9; two levels in R/NR: No-report, Report. *y*-axis: contrast of the Gabor grating; *x*-axis: conditions. ***B***, Comparison between the performances in the no-report (SD = 0.16) and report conditions (SD = 0.17) of each participant. *y*-axis: contrast of the Gabor grating; *x*-axis: conditions. SD, Standard deviation.

A significant main effect of R/NR (*F*_(1,38)_ = 45.35, *p *<* *0.001, η^2^ = 0.361) was observed (Table 3). There was no significant difference among the factors Levels and Times, and no interaction effect of any pair of factors. As for the incidental memory tasks, participants reported significant lower perceived contrast of the Gabor gratings after the no-report condition than that after the report condition. This result supported to our hypothesis that the participants experienced degraded contents of consciousness during the no-report condition. Figure 3*A* shows the boxplot of the performances under these three factors. Figure 3*B* shows the result by averaging all factors.

**Table 3:** Three-way ANOVA in experiment 1a

Source	df1	df2	*F*-ratio	*p*-value	η^2^
Times	2	38	0.292	0.749	0.008
Levels	1	38	1.177	0.285	0.016
R/NR	1	38	45.35	5.64e-08**	0.361
Times × Levels	2	38	0.596	0.556	0.016
Times × R/NR	2	38	0.316	0.731	0.008
Levels × R/NR	1	38	0.082	0.776	0.001
Times × Levels × R/NR	2	38	0.634	0.536	0.016

**p <* *0.01.

**Table 4:** 2 × 2 mixed ANOVA

Sources	df1	df2	*F*-ratio	*p*-value	η^2^
Levels	1	17	2.427	0.138	0.068
R/NR	1	17	4.631	0.046*	0.118
Levels × R/NR	1	17	0.615	0.444	0.017

*p < 0.05.

### Experiment 1b

**Figure 4.**
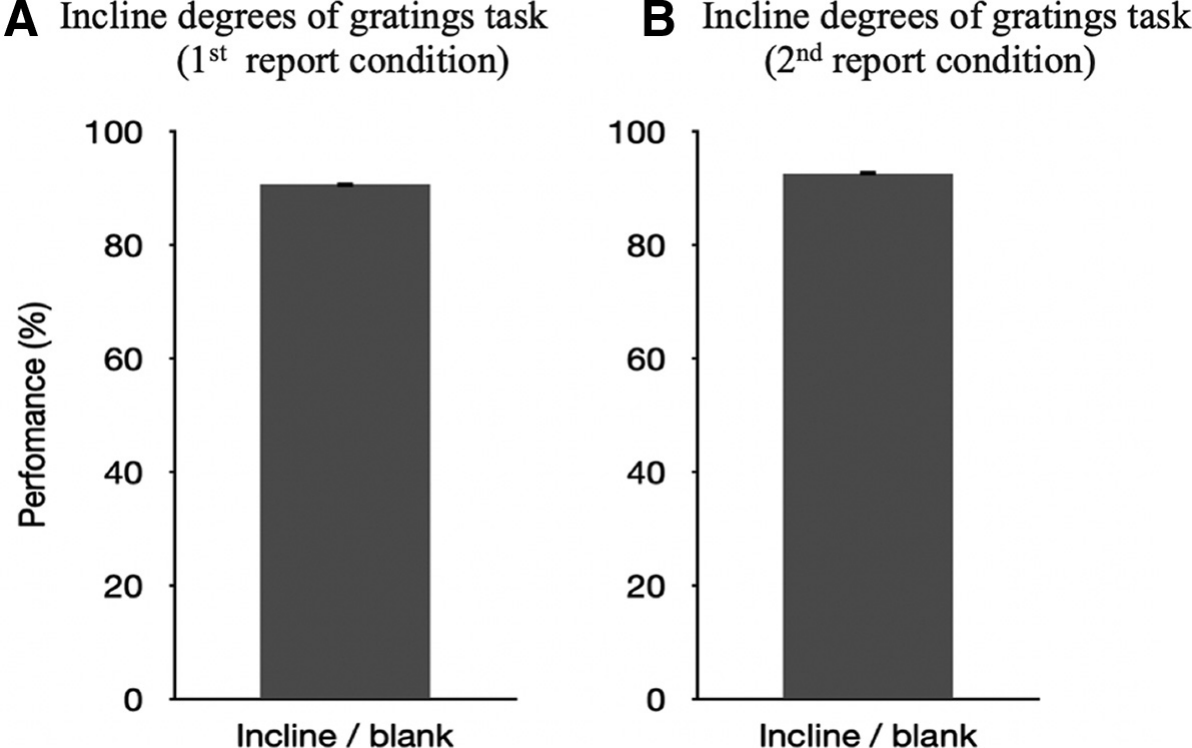
Results from both conditions in experiment 1b. In all plots, percentage correct (i.e., performance) is plotted on the *y*-axis. ***A***, Performance on the 45° incline/135° incline/blank task in the first report condition. The error bar indicates the SE (0.27%). ***B***, Performance on the 45° incline/135° incline/blank task in the second report condition. The error bar indicates the SE (0.28%). SE, Standard error.

In the first report condition, participants correctly reported what they saw on 90.6% (SD = 0.27%) of the trials (Fig. 4*A*). In the incidental memory task, the mean perceived contrast of the Gabor gratings was 0.534. In the second report condition, participants correctly reported what they saw on 92.5% (SD = 0.28%) of the trials (Fig. 4*B*). In the second incidental memory task, the mean perceived contrast of the Gabor gratings for was 0.584.

**Figure 5.**
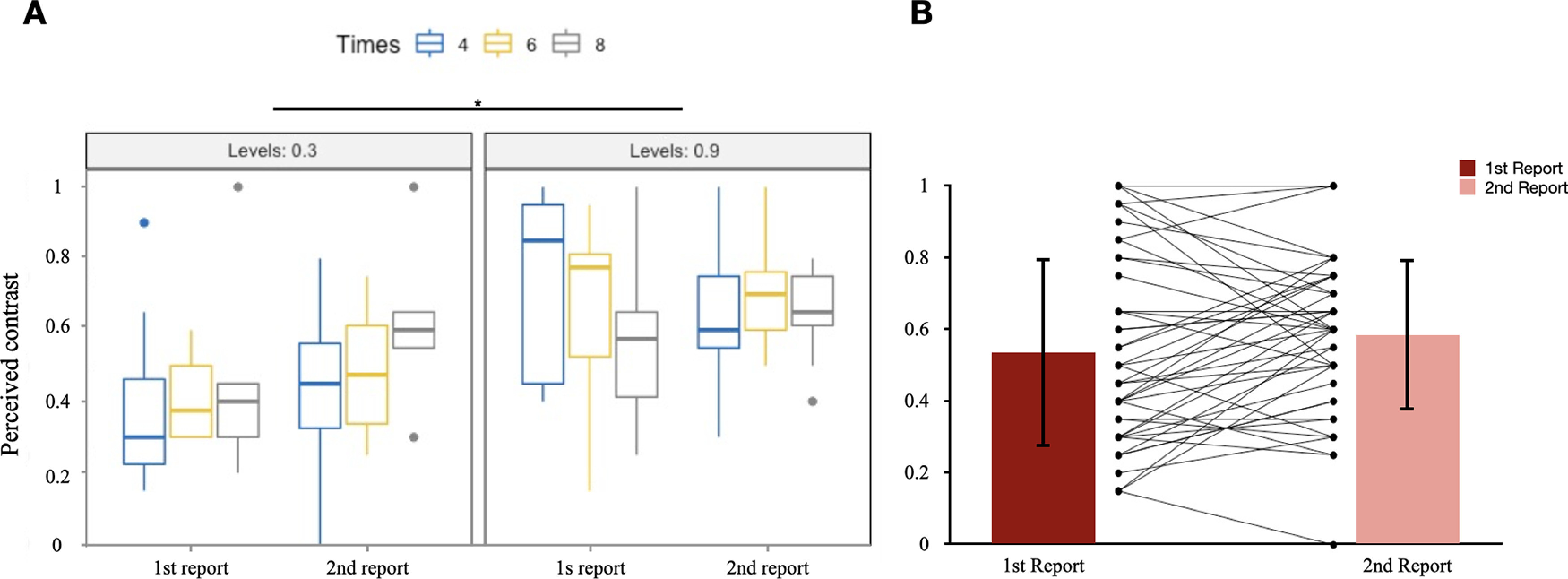
***A***, Perceived contrasts in the incidental memory tasks within all conditions. Three levels in Times: 4, 6, 8; two levels in Levels: 0.3, 0.9; two levels in R1/R2: first Report Condition, second Report Condition. *y*-axis: contrast of the Gabor grating; *x*-axis: conditions. ***B***, Comparison between the performances in the first report (SD = 0.26) and the second report (SD = 0.21) conditions of each participant. *y*-axis: contrast of the Gabor grating; *x*-axis: conditions. SD, Standard deviation.

There was no difference in perceived contrast (*F*_(1,38)_ = 2.689, *p *=* *0.109, η^2^ = 0.016, ANOVA) between the first and the second incidental memory task, suggesting that the results of experiment 1a were not caused by the order/learning effect. Figure 5*A* shows the box plot of the results and Figure 5*B* shows the result by averaging all factors.

### Experiment 2

#### Behavioral results

**Figure 6.**
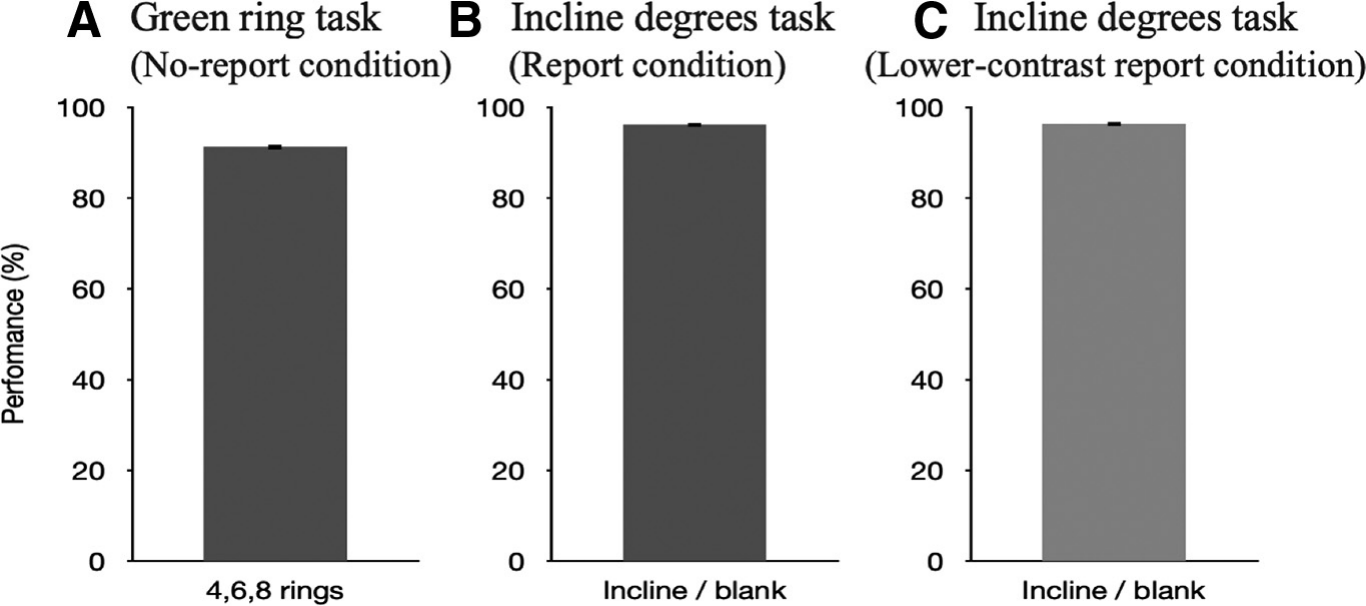
Results from all conditions in experiment 2. In all plots, percentage correct (i.e., performance) is plotted on the *y*-axis. ***A***, Performance on the green ring counting task in the no-report condition. The error bar indicates the SE (0.54%). ***B***, Performance on the 45° incline/135° incline/blank task in the report condition. The error bar indicates the SE (0.19%). ***C***, Performance on the 45° incline/135° incline/blank task in the lower-contrast report condition. The error bar indicates the SE (0.23%). SE, Standard error.

In the no-report condition, participants correctly reported seeing a green ring on 91.2% (SD = 0.54%) of trials (Fig. 6*A*). In the report condition, participants correctly reported seeing 45° incline Gabor gratings, 135° incline Gabor gratings, or blanks on 96.1% (SD = 0.19%) of trials (Fig. 6*B*). In the lower-contrast report condition, participants correctly reported seeing 45° incline Gabor grating, 135° incline Gabor grating, or blanks on 96.3% (SD = 0.23%) of trials (Fig. 6*C*).

**Figure 7.**
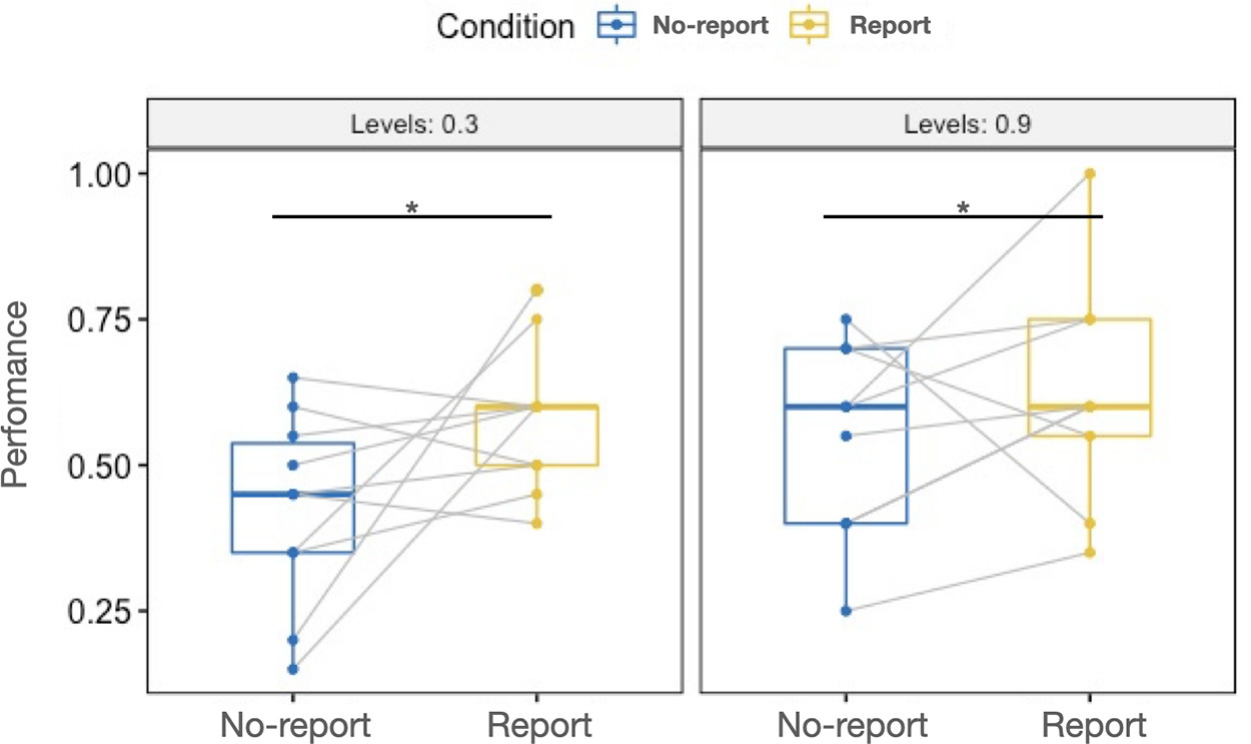
Boxplot with paired data; *p < 0.05. Two levels in Levels: 0.3, 0.9; two levels in Condition: no-report condition, report condition. *y*-axis: contrast of the Gabor grating; *x*-axis: conditions.

For the incidental memory tasks after the first and third phases, a 2 × 2 mixed design ANOVA was conducted (Table 4). Main effect of R/NR was observed (*F*_(1,17)_ = 4.63, *p *=* *0.046, η^2^ = 0.118, ANOVA). There was no significant difference between levels under the Levels factor (*F*_(1,17)_ = 2.427, *p *=* *0.138, η^2^ = 0.068, ANOVA), interaction effect of R/NR and Levels was not observed (*F*_(1,17)_ = 0.615, *p *=* *0.444, η^2^ = 0.017, ANOVA) either. Boxplot of the performances in these two factors was shown in Figure 7.

**Figure 8.**
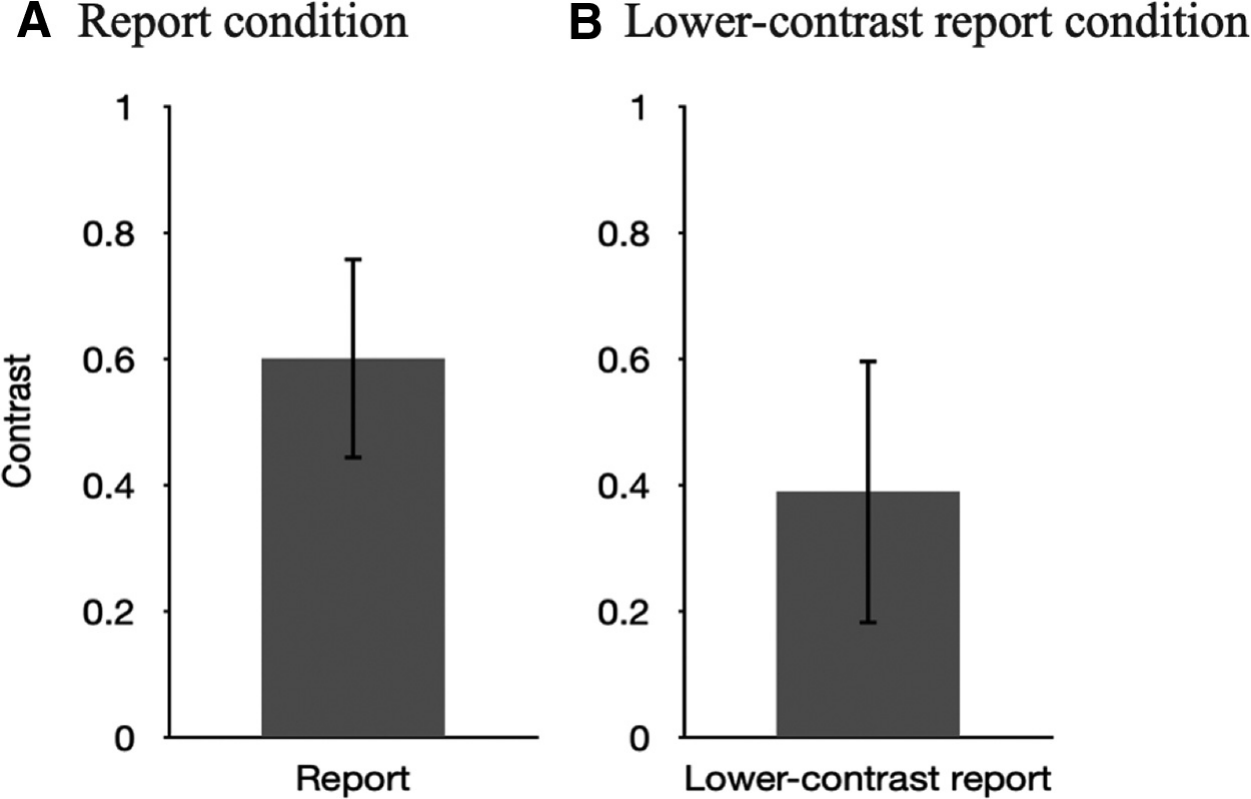
In both plots, reported contrast of the stimuli is plotted on the *y*-axis. ***A***, Average perceived contrast after the report condition. The error bar indicates the SE (0.21%). ***B***, Average perceived contrast after the lower-contrast report condition. The error bar indicates the SE (0.16%). SE, Standard error.

Since the stimuli in the lower-contrast report condition were different from the former two conditions, the results in the sixth phase were not included in the 2 × 2 ANOVA test. Instead, we directly compared the average perceived contrast in the sixth phase to that in the fourth phase. The perceived contrast in the sixth phase (average = 0.39, SD = 0.21) was significantly different from that in the fourth phase (average = 0.6, SD = 0.16), suggesting that the participants could accurately report contrast of the stimuli they saw in the report conditions (Fig. 8).

#### EEG results

##### Hypothesis-driven analyses of the P3b

**Figure 9.**
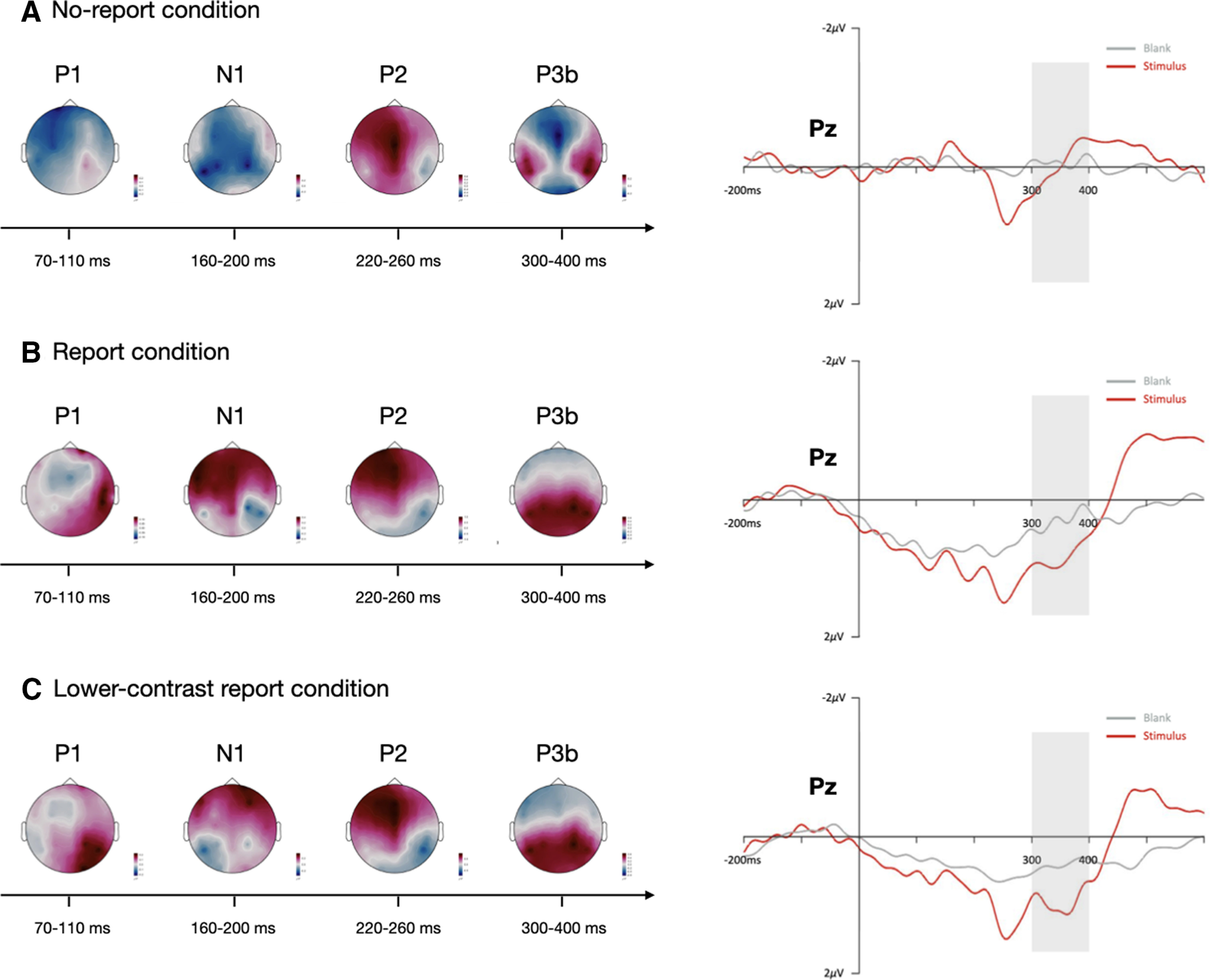
ERP results for no-report (top row; ***A***), report (middle row; ***B***), and lower-contrast report (bottom row; ***C***) conditions. For all conditions, topographical voltage distributions over a series of time windows and waveforms from a representative (Pz) electrode are plotted. Gray curves reflect the ERP of the critical stimuli, red curves reflect the ERP of blanks. Gray areas represent the ROIs of P3b. In both report and lower-contrast report conditions, clear P3b amplitudes could be observed during the 300- to 400-ms time window while critical stimuli were presented. However, the P3b amplitude vanished in the no-report condition regardless of whether the participants saw the stimuli or not. *p < 0.05. Amplitude scales for the topography maps are as follows: ***A***, ±0.3 μV (P1); ±0.4 μV (N1); ±0.7 μV (P2); ±0.4 μV (P3b); ***B***, ±0.15 μV (P1); ±0.4 μV (N1); ±1.0 μV (P2); ± 0.6 μV (P3b); ***C***, ±0.2 μV (P1); ±0.4 μV (N1); ±1.0 μV (P2); ±0.6 μV (P3b).

In the first phase and the third phase, we manipulated the reportability of the stimuli. In the third phase and the fifth phase, we manipulated the perceived contrasts of the stimuli. ERP results within the phases were summarized in Figure 9 by plotting voltage distribution maps (stimuli minus blanks) from a series of time windows over all electrode locations. The early visual ERP P1 was clearly present in both report conditions, while N1 was uniquely present in the no-report condition. The P2 component over the frontoparietal scalp was apparent in all of the three conditions.

In the first comparison, significant main effects of stimulus presence (*F*_(1,19)_ = 8.53, *p *=* *0.009, η^2^ = 0.073, ANOVA) and reporting task (*F*_(1,19)_ = 7.361, *p *=* *0.014, η^2^ = 0.152, ANOVA) were observed. The interaction between stimulus presence and reporting task was also significant (*F*_(1,19)_ = 13.36, *p *=* *0.002, η^2^ = 0.024, ANOVA). In the second comparison, only the main effect of stimulus presence (*F*_(1,19)_ = 21,39, *p *=* *0.0002, η^2^ = 0.114, ANOVA) was observed. There was no main effect of perceived contrasts (*F*_(1,19)_ = 2.16, *p *=* *0.16, η^2^ = 0.012, ANOVA), and the interaction between stimulus presence and perceived contrasts was not observed (*F*_(1,19)_ = 0.058, *p *=* *0.81, η^2^ = 0.00,006, ANOVA), either.

#### Data-driven analyses

**Figure 10.**
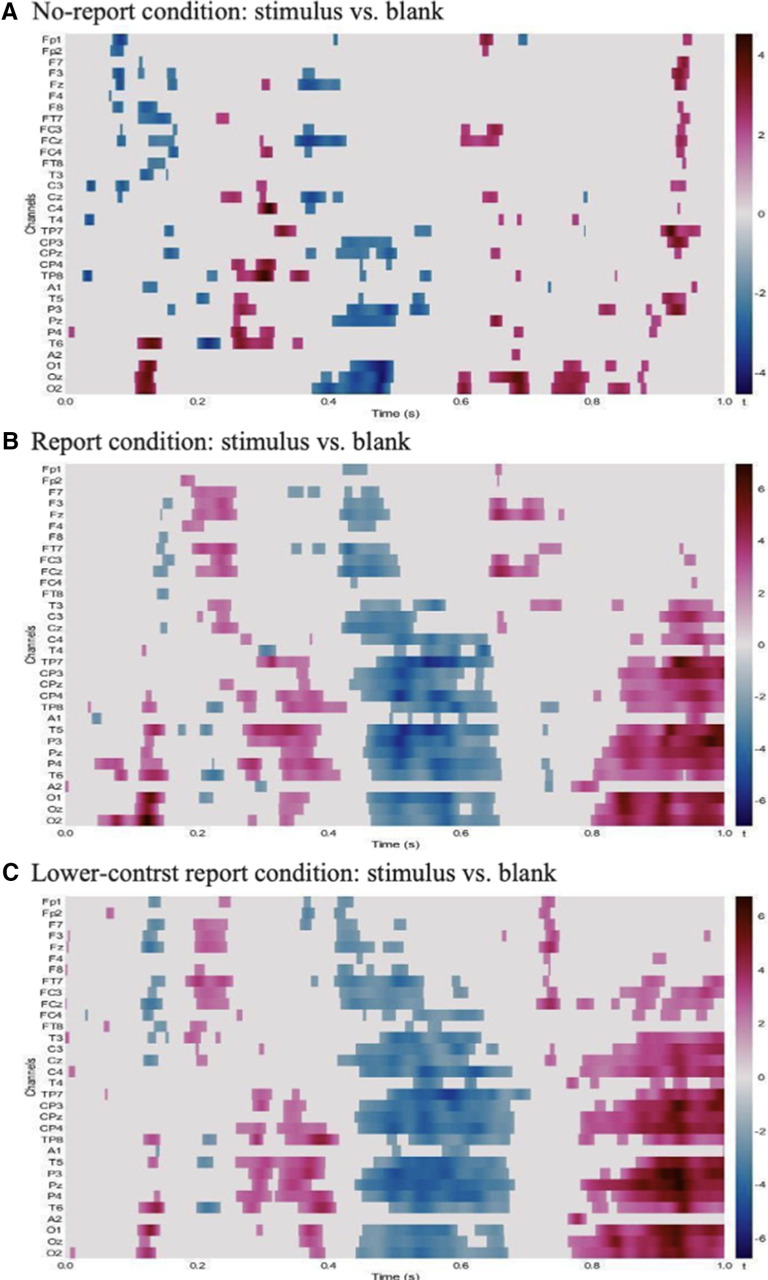
***A***–***C***, Results from the mass univariate analyses for all conditions. ***A***, No-report condition. ***B***, Report condition. ***C***, Lower-contrast report condition. Each individual electrode is plotted as a row on the *y*-axis, while time (in seconds) is plotted on the *x*-axis. Only significant t values (5% FDR) are plotted in this figure. FDR, False discovery rate.

In the no-report condition, amplitude differences were scattered and not as evident as the other two conditions. The differences concentrated at posterior P1, posterior P2, and anterior positivity around 900 ms. In both the report and the lower-contrast report conditions, amplitude differences were evident in the early (posterior P1; anterior P2) and late (posterior N400; posterior positivity, 800–100 ms) time windows. Unique to the report conditions, the P3b was evident from 300 to 400 ms, and the N400 was also evident from 400 to 600 ms (Fig. 10).

#### Exploratory analyses and source analyses

**Figure 11.**
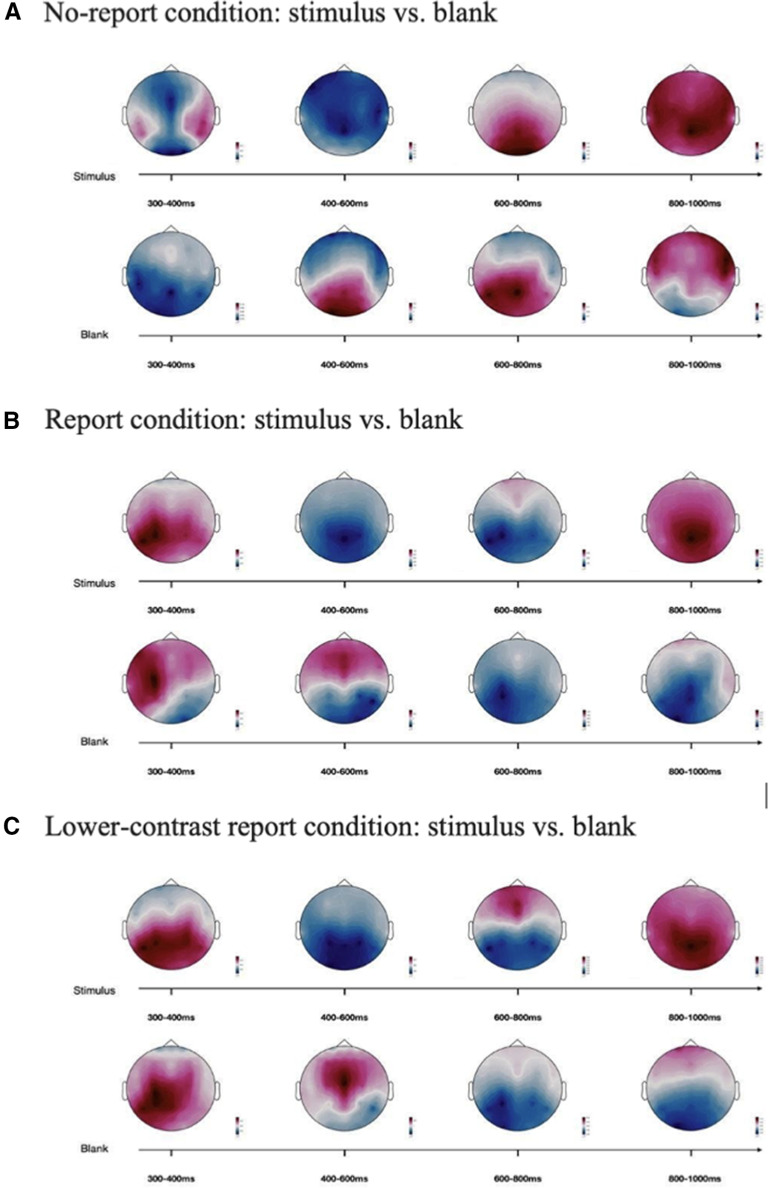
ERP differences during late time windows revealed by exploratory analyses for all conditions. The topographical differential voltage distributions over a series of time windows starting at 300 ms and ending at 1000 ms are shown for all the conditions. ***A***, No-report condition. ***B***, Report condition. ***C***, Lower-contrast report condition.

**Figure 12.**
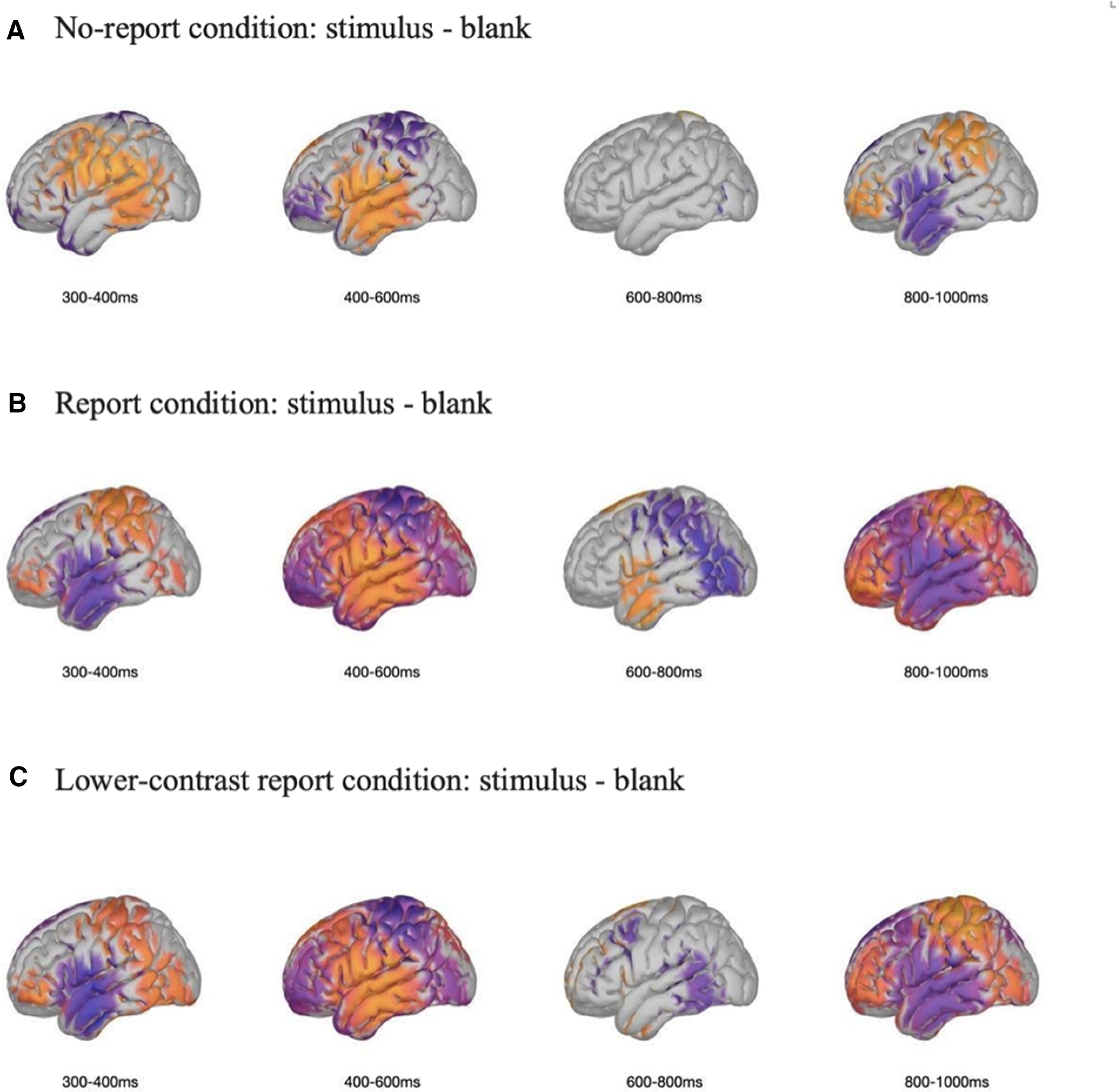
Source estimations of the differences between stimulus and blank for all conditions across four late time windows. ***A***, No-report condition. ***B***, Report condition. ***C***, Lower-contrast report condition. The ERPs of the blank were served as the baseline, only differences bigger than 2 × 10^−10^ voltage are plotted on the lateral surface of the Montreal Neurologic Institute brain. Areas plotted by orange color reflect amplitudes of the stimulus bigger than amplitudes of the blank. For those areas colored by purple, the amplitudes of the stimulus were smaller than blank.

After replicating the results of P3b in Cohen et al. (2020), we further conducted exploratory analyses (Fig. 11) and source analyses (Fig. 12) to investigate the nature of the P3b. Patterns of the source estimation were shown in Figure 12. Between 300 and 400 ms, there were clear decreases in source activity in the parietal lobe in the no-report condition relative to the other two conditions. Similar patterns in the parietal lobe could be observed between 800 and 1000 ms, and opposite patterns could be seen between 400 and 600 ms. For the frontal lobe, clear decreases in source activity in the no-report condition relative to the other two conditions could also be observed between 300 and 400 ms. Although source analyses from ERP data are imprecise and our experiment was not designed for optimized isolating sources, these findings could still be important as references for further investigations.

## Discussion

P3b, a subcomponent of P3, has been associated with a variety of perceptual and cognitive processes, including novelty detection in oddball paradigms (Squires et al., 1975; Duncan-Johnson and Donchin, 1977; Knight and Scabini, 1998; Barceló et al., 2002; Debener et al., 2005; Fischer et al., 2008), workload processing in dual-task paradigms (Matthews et al., 2006; Johannes et al., 2001; Kok, 2001), uncertainty resolution in signal detection tasks (Sutton et al., 1965; Squires et al., 1973), recallability in incidental memory tasks (Donchin et al., 1986; Fabiani et al., 1986; Azizian and Polich, 2007), reaction time and completion time in speeded tasks (Polich et al., 1983; Johnson et al., 1985; Emmerson et al., 1989; Pelosi et al., 1992a), expertise acquisition in learning tasks (Carrión and Bly, 2008; Seppänen et al., 2012; Morgan et al., 2016), etc. Some investigators also suggest P3b as a neural correlate of consciousness (Lamy et al., 2009; Dehaene and Changeux, 2011). However, this possibility has been challenged recently (Pitts et al., 2014; Shafto and Pitts, 2015; Cohen et al., 2020; Schröder et al., 2021).

In this study, we found that participants’ perceived contrasts of an identical stimulus were lower in the no-report condition than in the report condition. Furthermore, these different perceived contrast levels did not induce different P3b amplitudes. Hence, our finding still supports the claim that P3b is *not* a signature of perceptual awareness but is associated with postperceptual processing.

For the early components, P1/N1 showed very different patterns between the no-report condition and the two report conditions. However, both P1 and N1 components did not differ between the report condition and the lower-contrast report condition. This difference could be caused by participants paying more attention to the stimulus in the report condition. These results were consistent to the pattern of P3b in the corresponding three conditions. In addition, there is a large section of overlap time period between P2 and P3a. P3a usually has a maximum amplitude over frontal/central electrode sites, and appeared to be correlated with the habituation and target discrimination (Soltani and Knight, 2000; Polich, 2007). In this study, the P2 (or P3a) component was present only when the participants could see the stimuli, which is consistent with the previous findings that P3a is associated with target discrimination.

There are a few limitations in the current study. For example, there is a possibility that the participants actually experienced the same contents of consciousness in both the no-report and the report conditions: the different performances we observed were merely because of faster declination of memory when less attended. However, this possibility appears to be inconsistent with the robust phenomenon that attention can alter subjective visual perception and arguably a mediating factor of consciousness (Hsieh et al., 2005; Ling and Carrasco, 2006; Carrasco et al., 2008). Also, it remains unknown whether the mechanisms are same or different when lower perceived contrasts were induced by less distributed attention versus weaker stimulus strength. Future experiments are required to investigate these possibilities.

## Synthesis

Reviewing Editor: David Schoppik, New York University - Langone Medical Center Decisions are customarily a result of the Reviewing Editor and the peer reviewers coming together and discussing their recommendations until a consensus is reached. When revisions are invited, a fact-based synthesis statement explaining their decision and outlining what is needed to prepare a revision will be listed below. The following reviewer(s) agreed to reveal their identity: Michael Cohen. Note: If this manuscript was transferred from JNeurosci and a decision was made to accept the manuscript without peer review, a brief statement to this effect will instead be what is listed below.

Data presentation/analysis issues:

1. The exact determination of the contrasts is missing.

2. The EEG results are insufficiently presented. The different contrast on the early EEG components should be shown and an interaction with the P3. In any case, the early visual components should also be analyzed.

3. The study design not particularly well suited to determine visual contrasts in retrospect (memory bias etc.) It also became unclear to me how exactly this was done and why there was the over- and underestimation.

Suggestion for restructuring the manuscript:

I would suggest the authors group things by experiments. Right now they do Experiment 1 methods, Experiment 2 methods, Experiment 1 results, Experiment 2 results. I found this very very confusing. I would strongly suggest doing Experiment 1 methods, Experiment 1 results and then moving on to Experiment 2 in a similar way. As it was now, by the time we got to Figure 11, I had to scroll back several pages to much earlier in the manuscript to figure out what was going on.

How the data is shown for the first experiment is difficult and I had to read it several times to understand. I would strongly encourage the authors to simplify and clarify what they did and how they present the results. For example, when talking about the no-report condition data first, perhaps show us a) how often people got the green circle counting task right and then b) what the contrast values for the Gabor were in the incidental memory task. After doing that, then move on to the report condition data and show us a) how often they were correct with the main task (i.e., reporting) and then b) what the contrast values for the Gabor were in the incidental memory task. As it is now, the data are not reported in this simple way and it’s actually rather confusing what is getting reported (i.e., why does figure 2 only show data from the report condition?). If the order of the experiment was as it’s shown in Figure 1A, maybe show us the data that way? I’m not married to this idea, but as it is now is pretty confusing.

Relatedly, maybe put Figures 3 and 4 together since they are kind of just showing the same type of data? Perhaps even color code them in similar ways so a reader can group them across the figures? Figure 4 is basically just the averaged version of Figure 3 right? Am I missing something? The fact that they are separated and different colors made me think they must be different analyses?

When the authors started talking about the first and second report conditions, I was confused and had to go back and figure out what they meant by the first and second report conditions. Please be more clear about what those conditions are and why they are being separated, especially since the behavioral data in figure 5 is effectively identical anyways (also minor point, but in Figure 5A it says “1s report condition” and I think the authors mean “1st report condition”). Actually this extends more broadly since several figures after figure 5 are about first and second report conditions and I wasn’t always sure what they meant.

On page 12, all the sudden we’re talking about Experiment 2 and I was thinking “wait, what exactly counts as experiment 1? Is it just behavioral vs EEG data? No that can’t be right since on line 307, there is the phrase “Behavioral results” right there.” So where does one experiment begin and another end?

## Author Response

We thank both reviewers for the constructive comments on our manuscript. We believe that addressing these comments has made our manuscript clearer to our readers. Below please find our point-by-point responses to the reviewers.

The original comments are in green.

The responses are in black.

The revision made in the manuscript are in blue.

1. The exact determination of the contrasts is missing.

Resonse 1:

We have added in a description for how the contrast was.

Revision:

The contrast level was defined as how visible the stimulus is relative to the white background. The value should be a single float ranging 0.0 (transparent) to 1.0 (operant). Table 1 shows the corresponding relation between the Weber Contrast and the contrast defined in this study.

2. The EEG results are insufficiently presented. The different contrast on the early EEG components should be shown and an interaction with the P3. In any case, the early visual components should also be analyzed.

Resonse 2:

We have added voltage distributions over some specific series of time windows (P1, N1, P2) to show the early EEG components. Also, we have added the descriptions for the analyses of early components.

Revision:

Figure 11. ERP results for no-report (top row; A), report (middle row; B), and lower-contrast report (bottom row; C) conditions. For all conditions, topographical voltage distributions over a series of time windows and waveforms from a representative (Pz) electrode are plotted. Green curves reflect the ERP of critical stimuli, red curves reflect the ERP of blanks. Gray areas represent the ROIs of P3b. In both report and lower-contrast report conditions, clear P3b amplitudes could be observed during the 300-400ms time window while critical stimuli were presented. However, the P3b amplitude vanished in the no-report condition regardless of whether the participants saw the stimuli or not. * p < .05. Amplitude scales for the topography maps are as follows: A. {plus minus} 0.3μV (P1); {plus minus} 0.4μV (N1); {plus minus} 0.7μV (P2); {plus minus} 0.4μV (P3b); B. {plus minus} 0.15μV (P1); {plus minus} 0.4μV (N1); {plus minus} 1.0μV(P2); {plus minus} 0.6μV (P3b); C. {plus minus} 0.2μV (P1); {plus minus} 0.4μV (N1); {plus minus} 1.0μV (P2); {plus minus} 0.6μV (P3b).

The main pattern of results was visualized by plotting voltage distribution maps (stimuli minus blanks) from a series of time windows over all electrode locations. The early visual ERP P1 was clearly present in both report conditions, while N1 was uniquely present in the stimulus condition of no-report condition. The P2 component over the frontoparietal scalp was apparent in all of the three conditions.

Discussion:

For the early components, P1/N1 showed very different patterns between the no-report condition and the two report conditions. However, it could be clearly observed that both P1 and N1 components did not show differences between the report condition and the lower-contrast report condition. These results were consistent to the pattern of P3b in the corresponding three conditions. There is a large section of overlap time period between P2 and P3a. P3a usually has a maximum amplitude over frontal/central electrode sites, and appeared be correlated with the habituation and target discrimination (Polich, 2007; Soltana & Knight, 2000). In this study, the P2 (or P3a) component was present only when the participants could see the stimuli, which is consistent with the previous findings that P3a is associated with target discrimination.

3. The study design not particularly well suited to determine visual contrasts in retrospect (memory bias etc.) It also became unclear to me how exactly this was done and why there was the over- and underestimation.

Response 3:

We agree with the possibility that memory bias may affect the results. We actually have talked about this possibility in the discussion, please see the line 401 to line 406. Though this design cannot rule out the retrospective memory issue, it is in line with the evidence that lower attention could induce lower perceived contrast. And our data show that even when the participants perceived lower contrast, the P3b amplitude was not affected by this factor.

4. Suggestion for restructuring the manuscript

I would suggest the authors group things by experiments. Right now they do Experiment 1 methods, Experiment 2 methods, Experiment 1 results, Experiment 2 results. I found this very very confusing. I would strongly suggest doing Experiment 1 methods, Experiment 1 results and then moving on to Experiment 2 in a similar way. As it was now, by the time we got to Figure 11, I had to scroll back several pages to much earlier in the manuscript to figure out what was going on.

Response 4:

We have reconstructed the structure as suggested.

5. Relatedly, maybe put Figures 3 and 4 together since they are kind of just showing the same type of data? Perhaps even color code them in similar ways so a reader can group them across the figures? Figure 4 is basically just the averaged version of Figure 3 right? Am I missing something? The fact that they are separated and different colors made me think they must be different analyses?

Response 5:

Revised.

Revision:

Figure 3. A, Contrasts the participants adjusted to in the incidental memory tasks within all conditions. Three levels in Times: 4, 6, 8; two levels in Levels: 0.3, 0.9; two levels in R/NR: No-report, Report. Y-axis: contrast of the Gabor grating; X-axis: conditions. B, Comparison between the performances in the no-report (SD=0.16) and report conditions (SD=0.17) of each participant. Y-axis: contrast of the Gabor grating; X-axis: conditions.

Figure 5. A, Contrasts the participants adjusted to in the incidental memory tasks within all conditions. Three levels in Times: 4, 6, 8; two levels in Levels: 0.3, 0.9; two levels in R1/R2: 1st Report Condition, 2nd Report Condition. Y-axis: contrast of the Gabor grating; X-axis: conditions. B, Comparison between the performances in the 1st report (SD=0.26) and the 2nd report (SD=0.21) conditions of each participant. Y-axis: contrast of the Gabor grating; X-axis: conditions.

6. (i.e., why does figure 2 only show data from the report condition?)

Response 6:

We did not plot the figure for data from the no-report condition in experiment 1a because in every behavioral experiment, all the participants only had one chance to count how many times they saw the green ring. Hence, the data we got was only an average number, instead of plotting a diagram without an error bar, we directly described it in the context.

Revision:

In the revised version, we add a plot figure to visualize the result.

7. When the authors started talking about the first and second report conditions, I was confused and had to go back and figure out what they meant by the first and second report conditions. Please be more clear about what those conditions are and why they are being separated, especially since the behavioral data in figure 5 is effectively identical anyways (also minor point, but in Figure 5A it says “1s report condition” and I think the authors mean “1st report condition”). Actually this extends more broadly since several figures after figure 5 are about first and second report conditions and I wasn’t always sure what they meant.

Response 7:

We have added a description at the beginning of experiment 1b and clarified the procedure of experiment 1b.

Revision:

We conducted experiment 1b to investigate if there is a sequence effect that might influence participants’ performances in the two incidental memory tasks. Hence, in experiment 1b, the same report condition was presented twice to check if there was a sequence effect.

Visual stimulation and Experimental design

All stimuli and procedures were identical to those in experiment 1a except that there was no no-report condition in this experiment. Participants were asked to complete two phases of the report condition, and all of them completed the experimental procedure in a fixed order: (1) the first report condition with 64 trials; (2) an incidental memory task on the critical stimuli; (3) the second report condition with 64 trials; (4) an incidental memory task on the critical stimuli.

8. On page 12, all the sudden we’re talking about Experiment 2 and I was thinking “wait, what exactly counts as experiment 1? Is it just behavioral vs EEG data? No that can’t be right since on line 307, there is the phrase “Behavioral results” right there.” So where does one experiment begin and another end?

Response 8:

We have reconstructed the manuscript for clarification.
